# Ventricular septal rupture caused by myocardial bridge, solved by interventional closure device

**DOI:** 10.3325/cmj.2012.53.627

**Published:** 2012-12

**Authors:** András Zóka, Péter Andréka, Dávid Becker, Géza Fontos, Béla Merkely, György Szabó, András Szatmári, György Bárczi

**Affiliations:** 1 Semmelweis University Heart Center, Budapest, Hungary; 2 Gottsegen György Center of Cardiology, Budapest, Hungary

## Abstract

Myocardial bridging is a common coronary anomaly, which is generally described as a benign phenomenon. However, a growing number of studies consider this anomaly a relevant pathophysiological phenomenon with serious pathological consequences. Here we report on the case of an 88-year-old woman suffering from myocardial infarction and ventricular septal rupture, lacking any recognizable coronary disease except for a myocardial bridge causing the systolic compression of the left anterior descending coronary artery. A wide range of diagnostic procedures, including coronarography, echocardiography, and magnetic resonance imaging were used. The septal rupture was finally closed by using a percutaneous closure device. This event indicates that myocardial bridges – at least in some cases – may have notable clinical relevance.

Some epicardial coronary arteries may have an intramyocardial segment referred to as a myocardial bridge (MB). This condition is generally described as a benign vascular anomaly. However, in some cases it has been reported to cause acute coronary syndrome, malignant ventricular arrhythmia, and sudden cardiac death (1). Research based on coronary angiography, a gold standard for diagnosing myocardial bridging, reports that this anomaly occurs in 1.5%-16% of members of Caucasian population (2). On the other hand, pathologists find myocardial bridging in up to 80% of all examined hearts (1). The relevance of the limitation of myocardial perfusion by compression of the anomalous vessel during the systole is often brought into question. At the same time, some studies report relevant asynergy in the contraction of the bridge during the diastolic blood flow (1). It has been well established that the proximal segment of the MB is more prone to atherosclerosis, while the tunneled coronary segment is spared (1). Plaques within the distal segment also seem to be less frequent (3). This is likely to be related to the higher proximal and lower distal intracoronary pressure-oscillation, which also implies shear stress (4). The tunneled segment is described as being prone to vasospasm (1).

## Case report

A 88-year-old female patient was hospitalized with chest pain lasting for 20 hours and a previously unknown left bundle branch block on the ECG. Her medical history revealed previous gastro-esophagal reflux disease associated with hiatus hernia, duodenal ulcer, bronchial asthma, mammary fibroadenoma, macular degeneration, cataract, vertebrobasilar syndrome, cervical and lumbal spondylosis, fibromyalgia, osteoporosis, essential tremor, and psychiatric disorders (depression, dysthimia, and maladaptive anxiety). The patient had a successful sports career as a fencer in her youth.

**Figure 1:**
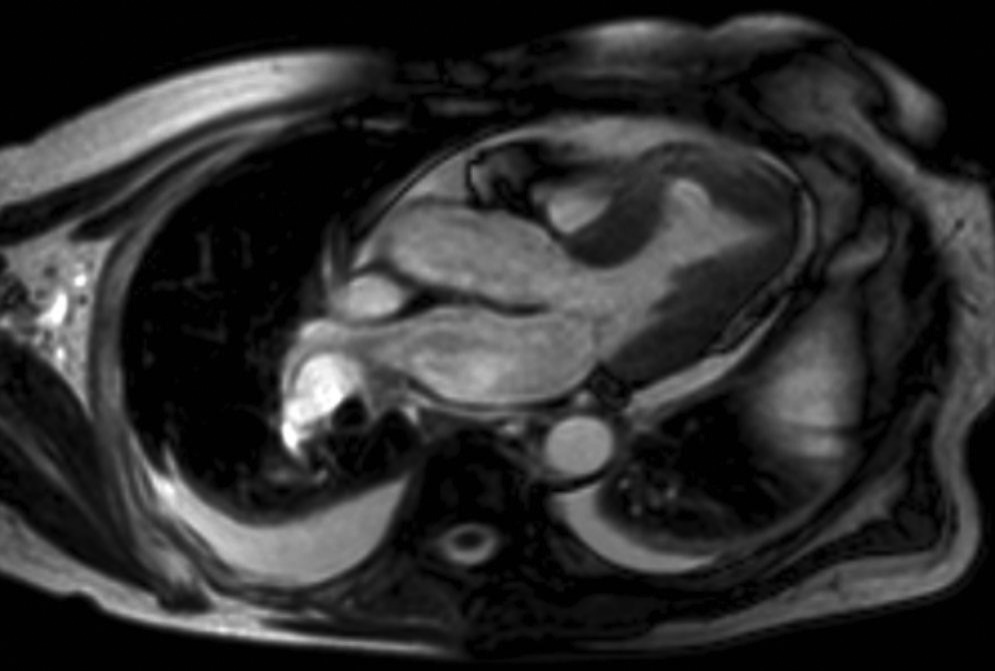
The aneurism containing the rupture can be seen on the distal septum (MRI image).

**Figure 2:**
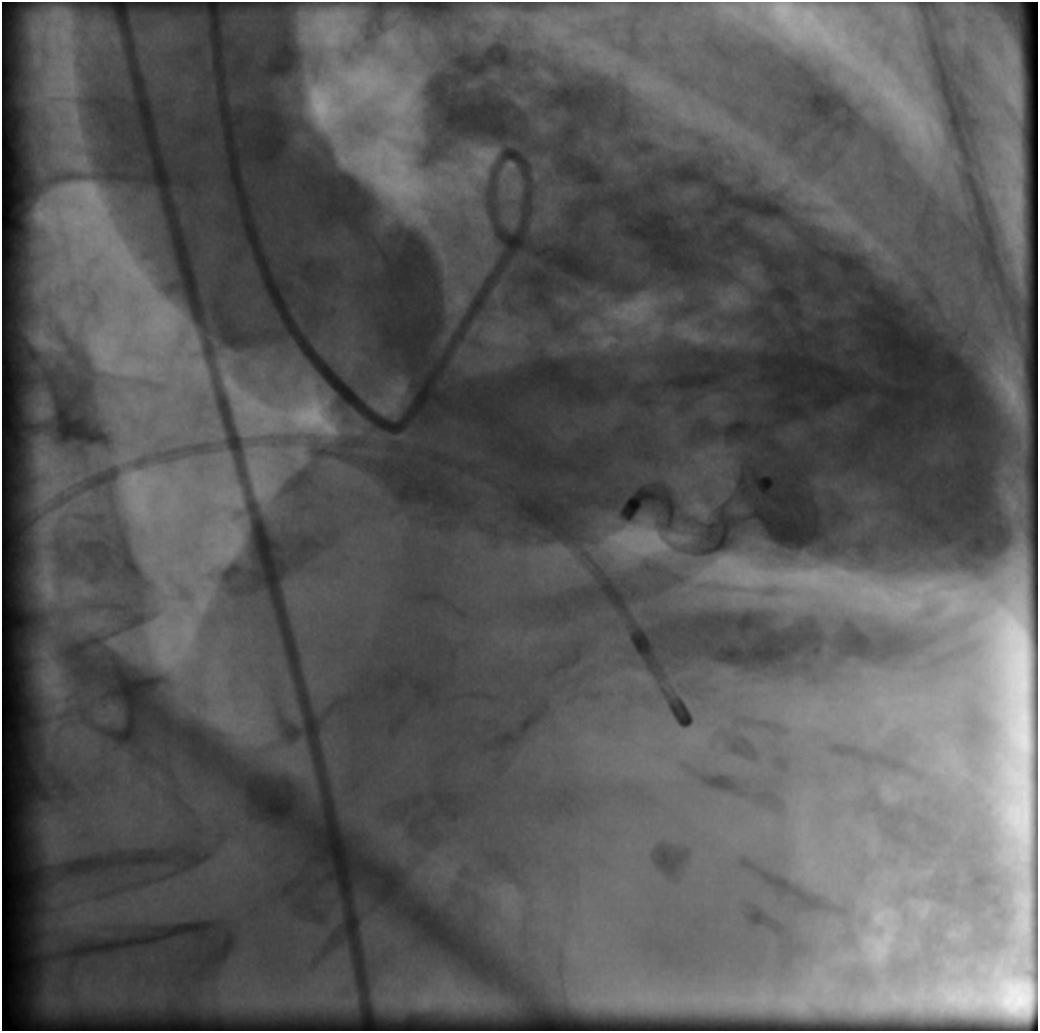
The device is placed in the rupture, leaving only minimal leakage behind.

Coronary artery stenosis was ruled out with urgent coronarography, but a myocardial bridge 19.11 mm in length was described on the middle third of the left anterior descending coronary artery. The minimum systolic lumen diameter of the bridge was 0.91 mm and the maximum systolic lumen reduction of the diastolic diameter was 82.78%. On account of the lack of any fixed stenosis as possible target of dilatation and/or stent implantation, an intervention was not performed. According to literature, the benefits of stenting, as possible treatment for myocardial bridging are not obvious, even stent fracture was described and symptoms might often reoccur (5,6). Myocardial bridging can be classified into three levels of severity based on systolic vessel compression: below 50% (grade I), between 50 and 75% (grade II), and above 75% (grade III) reduction of diastolic diameter. In cases of symptomatic grade III myocardial bridging, some authors recommend a surgical approach (myotomy or bypass grafting, both carried out via open heart surgery). In symptomatic grade II bridging, pharmacological reduction of the heart rate seems to be the best choice (7). Our case could be described as grade III according to the angiography, but considering all the circumstances (among others: age, comorbidities, non-life-threatening condition, as well as the long asymptomatic history) and the risk-benefit ratio of such an intervention, it did not come into consideration. Laboratory results revealed positive cardiac necroenzymes with the highest creatine kinase-MB being 77 U/L. Echocardiography showed a 22 × 15 mm aneurysm on the distal third of the anterior septum, in which 8-mm wide ventricular septal rupture (VSR) jet was detected (peak gradient: 100 Hgmm). Some pericardial fluid was detected as well. The described signs led to the diagnosis of a myocardial infarction. The maximum left ventricular ejection fraction (EF) was 60%. To clarify the relation between the infarction and the defect we carried out MRI, which showed colocalization of the late contrast enhancement and the VSR (Figure 1). The dilated right ventricle and the relatively high left ventricular EF were also confirmed. Considering the significant discrepancy between the right and the left ventricular cardiac output (pulmonary-systemic flow ratio, Qp/Qs-:1.75), we opted to close the rupture. As open heart surgery might impose a possibly fatal burden on an old age patient, a transcatheter obturation approach was decided upon. Left ventricular angiography was carried out by a right femoral artery approach. A Terumo guidewire (Terumo Europe N.V, Leuven, Belgium) was driven through the ventricular septal defect into the right ventricle, right atrium, and vena cava superior. An arteriovenous loop was formed with a Multisnare (pfm medical ag, Köln, Germany) catheter and 9F sheath was inserted from the right jugular vein through the defect. Through the sheath the defect was closed with a 12 mm AMPLATZER® (AGA Medical Corporation, North Plymouth, MN, USA) muscular ventricular septal defect occluder. Despite the right ventricular disc malalignment, the position of the device was stable. The intervention was successful and after the procedure only minimal residual flow was detected through the device (Figure 2).

## Discussion

In this case, a clear pathological or pathophysiological condition underlying the myocardial infarction and the ventricular rupture was not established. We found no visible atherosclerotic plaques and no vasospasm was noted during coronary angiography. Some data suggest proximal clot formation as a mechanism for bridge obstruction, even postulating its transient repetition followed by spontaneous lysis (8). Nevertheless, in this case the diagnostic procedures showed only myocardial bridge anatomy and aneurism containing the rupture.

The VSR is a relatively rare mechanic complication of myocardial infarction and an especially rare complication of heart attack caused by MB. Therefore, we also considered the possibility of this VSR being a congenital defect. However, there are a few arguments against this theory:

• A long lasting 1.75 times higher right ventricular cardiac output would have caused visible ventricular hypertrophy, which was not found.

• Although the distal septal area is a possible localization for a congenital defect, this wall segment showed characteristics typical of ischemia: hypokinesis, normal thickness, and delayed contrast hyperenhancement.

• A congenital jet normally crosses the wall perpendicularly, while in our case it crossed the wall diagonally.

• It is well known that small congenital defects affecting the muscular septum tend to close spontaneously until the age of 7, but the one presented in our study remained open despite its small size (9).

These arguments, the lack of significant stenosis or any other visible coronary anomaly, the localization of the infarction, and the normal left ventricular ejection fraction led us to the conclusion that the myocardial bridge played a significant role in the patient’s ventricular septal rupture. However, it is still unclear why this bridge did not cause any disruption before the patient’s advanced age. Due to this, we cannot certainly identify the bridge as the cause of this acute coronary syndrome. In our opinion, it amplified the vulnerability of the affected myocardium.

Recent exposure of the patient to stressful family events might have also been a part of the multifactorial risk pool, which can lead to a myocardial infarction. The patient also mentioned occasional chest pain in the pre-infarction period, self-treated with sublingual nitrate. As nitrates are known to impair the hemodynamic properties of the tunneled segment, this could have also been related to the described pathological event (1).

This case underlines the clinical significance of myocardial bridging and encourages us to consider it as a serious malformation that should be recognized and dealt with as it might occupy a role in the process leading to acute coronary syndrome.
